# Perceptions of success of a local UK public health collaborative^†^

**DOI:** 10.1093/heapro/dav088

**Published:** 2015-08-27

**Authors:** H. J. Littlecott, K. R. Fox, A. Stathi, J. L. Thompson

**Affiliations:** 1 Centre for the Development and Evaluation of Complex Interventions for Public Health Improvement (DECIPHer), School of Social Sciences, Cardiff University, Cardiff, UK; 2 School of Sport, Exercise & Rehabilitation Sciences, University of Birmingham, Birmingham, UK; 3 Centre for Exercise, Nutrition and Health Sciences, University of Bristol, Bristol, UK; 4 Department for Health, University of Bath, Bath, UK

**Keywords:** academic–practitioner collaboration, collaborative, network, older adults, physical activity

## Abstract

Successful public health initiatives require multi-sector collaboration. AVONet was a UK collaborative developed to provide evidence-based strategies for active ageing. This study explored the success of AVONet in the achievement of its objectives as perceived by all partners. A convergent parallel mixed-methods design was employed, utilizing a quantitative survey and qualitative semi-structured interviews. Data collection was undertaken in September 2010, 18 months after establishing the collaborative and 6 months after funding had ceased. AVONet partners (*n* = 24) completed a 27-item survey. A sub-sample of four academics and four practitioners participated in semi-structured interviews. Quantitative and qualitative comparisons were made between academics' and practitioners' perceptions of success, potential for sustainability and satisfaction with structure and relationships. Participants perceived the AVONet collaborative positively. Significant between-group (academic v practitioner) differences in survey responses were observed for success (*U* = 19.5; *p* = 0.003) and structure (*U* = 125.5; *p* = 0.001). Strong positive correlations were observed between success and structure and balance between information transfer and exchange (*r* = 0.756; *p* < 0.001). Interviews confirmed positive perceptions and perceived importance of the collaborative and highlighted the need for further integration and tangible outcomes for practitioners. Suggestions to enhance sustainability were provided, such as smaller working groups and local council-led governance. Perceived success in building a multi-sectoral collaborative can be achieved during a 10-month period, despite differing needs of contributors. For collaboratives developed as a result of external funding aimed primarily at facilitating research, involvement of practitioners at an early stage may help set more comprehensive goals, supportive communication strategies, and increase potential for sustainability.

## BACKGROUND

Public health research funders in the UK increasingly emphasize the importance of collaboration between academics and practitioners in order to enhance the relevance and applicability of research (Kelly and McNicoll, 2011). Public health initiatives are increasingly devolved to localities in order to meet local and regional needs and to be responsive to local characteristics (Rummery and Coleman, 2003). For such programmes to fulfil their potential they require contributions and collaboration of different agencies (Asthana *et al.*, 2002; Stathi *et al.*, 2012). For example, research has indicated that collaborative working focusing on older adults is associated with more successful levels of funding (Glendinning, 2003), enhancement of shared principles, knowledge and understanding (Leutz, 1999) and improvements in inter-organizational relations (Rummery and Coleman, 2003). Collaboration between academics and practitioners has been shown to be beneficial in terms of establishing joint governance, implementing interventions (Akintobi *et al.*, 2014) and increasing the quality and quantity of evidence-based practice (Neri *et al.*, 2014). Although diversity can aid successful collaboration, managing and coordinating diverse collaborations whilst avoiding tension and frustration presents a major challenge (Lasker *et al.*, 2001), thus highlighting the need to explore determinants of successful collaborations as perceived by different types of stakeholders.

Structuration Theory may be utilized to understand collaborations as it posits a complicated and reciprocal relationship between structure and agency, whereby individual actors have active agency which can reproduce or change social structure (Giddens, 1984; Greenhalgh and Stones, 2010). According to Structuration Theory, structure and agency should not be studied in isolation due to their interdependency, as one cannot be fully understood without the other (Giddens, 1984). Structure is not simply defined as the pattern of ties between actors, it represents the context of social agency as an outcome and mediator, not as a detached phenomenon (Giddens, 1984). This provides a useful theory to guide research aiming to understand how elements of collaborative working are perceived to be successful by contributors.

The Avon Network for the Promotion of Active Ageing in the Community (AVONet) was a public health collaborative, based in the southwest of England, UK. It was initially funded for 10 months by the Lifelong Health and Well-Being (LLHW) research initiative (managed by the Medical Research Council), with the aim of establishing a sustainable collaborative. The funding (£48 058) supported the organization of the activities planned by the collaborative, including meetings, three workshops, the invitation of national and international academics with expertise in the promotion of active ageing, administrative support and a part-time research associate post. AVONet partners included university academics, health professionals, local authority service providers, charitable trust employees, volunteers and older adults. All partners participated unpaid in the collaborative supplementary to their existing job role, although travel expenses were reimbursed. AVONet was initially set up through funds granted to the academic partners, with the subsequent invitation to practitioners to contribute. Therefore, the aims and objectives of the AVONet were developed by academics and shaped by the terms of the funding.

The purpose of this study was to gain an understanding of the degree to which two different types of AVONet partners—(a) academics and (b) practitioners (e.g. health professionals, local authority service providers, charitable trust employees and volunteers)—perceived that the collaborative had functioned successfully to achieve its specific objectives. These AVONet objectives, which were set by academics for funding purposes, were (i) holding collaborative meetings; (ii) reviewing evidence on effectiveness of physical activity (PA) promotion initiatives; (iii) re-analysing and collating existing data; (iv) comparing approaches to PA promotion against evidence-based criteria; (v) holding effective focus groups; (vi) producing recommendations for PA promotion for older people; and (vii) preparing a research proposal.

The specific research questions addressed in this article were as follows: what was the degree to which (i) AVONet was perceived by its partners to be successful/unsuccessful in achieving its aims and objectives?, (ii) the structure and partner interactions built within the collaborative were related to its perceived success/lack of success?, (iii) the AVONet was seen to be sustainable? and (iv) there was a difference in these perceptions between academic and practitioner partners?

## METHODS

A convergent parallel mixed-methods design was employed, whereby a survey and semi-structured interviews were conducted concurrently to investigate the perceived success of AVONet as a collaborative. The results from each method were assessed and then compared to ascertain the extent to which they substantiated each other (Creswell and Plano Clark, 2011). This provided more comprehensive sources of information on themes and offered the opportunity to confirm, support or reject findings (Creswell and Plano Clark, 2011). Data collection was undertaken in September 2010, 18 months after establishing the collaborative and 6 months after funding had ceased. Ethics approval for this study was obtained from the University of Bristol. This research was conducted as part of an MSc degree, and data collection, analyses and interpretation were supervised by an academic (J.L.T.) after the completion of the AVONet project.

### Sample and recruitment

The AVONet contributors were originally recruited via a region-wide invitation, which was extended to all leading organizations with a focus on active ageing. Involvement over a 10-month period included reviewing materials, participation in discussion groups, and three workshops. University academics (*n* = 14) were selected to provide diverse academic perspectives on ageing including primary care, public health, psychology, sociology, transport, as well as PA and were involved in the proposal for funding for the collaborative. Once funded, practitioners were invited to join the collaborative and five health professionals, 13 local authority service providers and 16 charitable trust employees and volunteers enrolled. This resulted in a total of 48 AVONet contributors.

### Survey

An anonymous online survey was designed (Giddens, 1984) using the framework of Structuration Theory. It was constructed to assess partners' perceptions of four themes regarding AVONet: the degree of *success* (or lack of) in achieving its aims and objectives, and the degree of success of its *structure, interactions* (*agency*), and *sustainability* for achieving its objectives*.* The survey was designed specifically for the current study as, at the time of data collection, the only existing measures such as the Index of Interdisciplinary Collaboration (Bronstein, 2002) were either based upon collaboration in clinical settings with patients or were based upon community settings. The authors felt that these measures did not allow the level of specificity and relevance for addressing the concepts and constructs, which were the focus of this study. We are aware that more relevant measures have been developed since, such as the Coordinated Action Checklist (Wagemakers *et al.*, 2010).

The survey included 23 items, which were set out in a five-point Likert format from ‘strongly disagree’ to ‘strongly agree’ plus a ‘don't know’ option. General questions were asked to ascertain participants' age, gender, organization of employment and join date. Level of success was defined as the degree to which participants agreed that AVONet aims and objectives (outlined in ‘Background’ section) were achieved (Provan and Milward, 1995). Eight survey items focused upon overall perceptions of success and the extent to which participants felt that the AVONet had achieved each of these objectives.

Structure was defined as the constituency of collaborative groups and their connectedness, as perceived by the actors (Greenhalgh and Stones, 2010). The groups were the core management group (CMG) of academics, other research co-applicants and collaborators (research, local authority and healthcare), an advisory group of practitioners (local authority, health care and non-governmental organizations) and older adult service users. Through seven survey items, participants were asked to state the degree to which this structure had successfully contributed to each of the collaborative aims and objectives and their level of satisfaction with the structure. These seven items were then combined into one item for analysis, representing overall satisfaction with collaborative structure. Four further items addressed: (i) the perceived degree of interdisciplinarity of collaborative partners; (ii) the perceived contribution of collaborative structure to aims and objectives of the organization the participant worked for; (iii) the perceived contribution of collaborative structure to aims and objectives of AVONet and (iv) the perceived impact of personal actions on collaborative structure.

Interactions were defined as the balance between information transfer and exchange, transfer being one-way delivery of information, and exchange involving receiving and contributing information (Giddens, 1984). Through a single item, participants were asked to state the degree to which AVONet offered an appropriate balance between information transfer and exchange.

Sustainability was defined as the potential for AVONet to continue to provide a useful function and for collaborative partners to build stable, long-term relationships following the termination of the research grant (Provan and Kenis, 2008). Three items assessed the extent to which participants agreed that sustaining AVONet would be beneficial for: increasing PA levels in older adults; promoting ageing research; and helping achieve their organizations' aims.

The survey was first piloted with AVONet partners, which led to the order and wording of some questions being changed. An email invitation to the final version of the survey, which took ∼20 minutes to complete, was sent to all 48 AVONet contributors. The initial email was followed by up with three reminder emails at 2-week intervals to encourage participation.

### Interviews

Individuals were purposively sampled from the group of AVONet contributors to participate in a semi-structured interview. They ranged in age and experience and were selected from different levels within the collaborative structure (CMG, co-applicants, collaborators, advisory group). Interviews were conducted by the lead researcher (H.J.L.) at the participants' place of work or in a public setting, such as a café, of the participants' choice and lasted up to 30 min.

The themes and questions for the interview schedule mirrored the themes of the survey, exploring them in more detail (Giddens, 1984; Hsieh and Shannon, 2005). All interviews were transcribed verbatim and accuracy of transcription was checked by a second independent researcher. Participant/organization names were removed to ensure anonymity.

### Analysis

Survey responses were not normally distributed and, therefore, non-parametric descriptive statistics were applied. Mann–Whitney *U*-tests were used to compare the distribution of responses from practitioners and research partners for each item and overall distribution for each group where there were several items. Spearman's correlations were used to assess the relationships between perceived success, structure, interactions and sustainability. Significance was set at *p* < 0.05. All statistical analyses were performed using SPSS version 16.0 (SPSS Inc., Chicago, IL).

Directed content analysis (Hsieh and Shannon, 2005) using a naturalistic paradigm was used to interpret the interview transcripts. A naturalistic paradigm states that there are multiple subjective realities and that these are socially constructed (Lincoln and Guba, 1985). First, during the content analysis, the content of the original themes developed using Structuration Theory was analysed and interpreted in the context of relevant research literature. During this process, emerging themes, codes or subcategories were also created and labelled (Weber, 1990). The original and emerging codes/themes from the qualitative analysis are listed in Table 1.

**Table 1:** Original and emergent interview themes and sub-themes

Original themes	Emergent themes	Emergent sub-themes
Success	Successful	Own opinion
		Opinion of others
	Not successful	Own opinion
		Opinion of others
Structure	Effect of actions on structure	Positive
		Negative
	Effect of structure on actions	Positive
		Negative
	Effect of structure on achievement of aims	Positive
		Negative
	Satisfaction	Positive
		Negative
Interactions	Good balance between information transfer and exchange	Own opinion
		Opinion of others
	Imbalance between information transfer and exchange	Own opinion
		Opinion of others
Sustainability	The collaborative should be sustained	Own opinion
		Opinion of others
	The collaborative should not be sustained	Own opinion
		Opinion of others

## RESULTS

A 50% response rate to the survey included 11 researchers and 13 practitioners, with an equal number of participants in each of the following age groups; 26–35, 36–45 and 56–65. There were no significant differences between groups for age (*U* = 68.5; *p* = 0.857) or gender (*U* = 65.5; *p* = 0.658).

Out of 12 purposively selected individuals, 8 agreed to participate in an interview. These were four academics and four practitioners from city councils, primary care trusts and the charitable trust sector. Six interviewees were 25–45 years old and two >56 years, all of whom had also completed the survey.

### Success

Participants provided an average of 75% positive responses (see Table 2). Overall, academics' responses were significantly different to practitioners' (*U* = 19.5; *p* = 0.003). Academics provided 91% and practitioners 62% of responses either in the ‘agree’ or ‘strongly agree’ categories. Mann–Whitney tests revealed significant differences for overall feelings (*U* = 113.5; *p* = 0.013) and perceptions of whether existing local data were analysed (*U* = 105.5; *p* = 0.047), whether ‘best bet’ options for PA promotion were produced (*U* = 120.5; *p* < 0.001) and whether a research proposal was produced (*U* = 108.0; *p* = 0.035).

**Table 2:** Positive responses and group differences for each survey section

	Overall	Academics	Practitioners	*p* value for group difference in overall responses	Mann–Whitney *U*
	Number/24 (%) of positive responses^a^	Number/11 (%) of positive responses^a^	Number/13 (%) of positive responses^a^		
Success in achieving AVONet aims and objectives (eight items)					
Overall feelings	24 (100)	11 (100)	13 (100)	0.013*	113.5
Held series of meetings with multidisciplinary contributions	20 (83.3)	10 (90.9)	10 (76.9)	0.277	91.0
Synthesized the existing evidence	17 (70.8)	9 (81.8)	8 (61.5)	0.424	85.5
Analysed existing relevant local qualitative and quantitative data	18 (75.0)	10 (90.9)	8 (61.5)	0.047*	105.5
Compared options for physical activity promotion against evidence and feasibility criteria	17 (70.8)	9 (81.8)	8 (61.5)	0.207	93.5
Incorporated service user views through workshops and focus groups	17 (70.8)	10 (90.9)	7 (53.9)	0.082	101.5
Synthesized all information to produce a set of ‘best bet’ options for activity promotion	15 (62.5)	11 (100)	4 (30.8)	<0.001**	120.5
Prepared at least one substantial research proposal for evaluation of a ‘best bet’ physical activity promotion programme	19 (79.2)	10 (90.9)	9 (69.2)	0.035*	108.0
For all success items	18.4 (76.7)	10 (90.9)	8 (62.3)	0.003**	19.5
Structure (five items)					
Overall satisfaction with structure	11 (45.8)	8 (72.7)	3 (23.1)	0.011*	114.5
Degree of interdisciplinarity	18 (75.0)	10 (90.9)	8 (61.5)	0.013*	114.0
Contribution to meeting AVONet aims and objectives	14 (58.3)	7 (63.6)	7 (53.9)	0.134	97.5
Contribution to meeting your organization's aims and objectives	14 (58.3)	9 (81.8)	5 (38.5)	0.035*	107.0
Influence of personal actions on AVONet structure	7 (29.2)	4 (36.4)	3 (23.1)	0.041*	107.5
For all structure items	12.8 (53.3)	7.6 (69.1)	5.2 (40.0)	0.001**	125.5
Information exchange (one item)					
Good balance between transfer and input from partners	15 (62.5)	9 (81.8)	6 (46.2)	0.134	97.5
Benefits of sustainability (three items)					
For physical activity of local people	18 (75.4)	10 (90.9)	8 (61.5)	0.106	99.5
For research with the AVON area	17 (70.8)	9 (81.8)	8 (61.5)	0.494	84.0
For the achieving the aims of you organization	18 (75.0)	9 (81.8)	9 (69.2)	0.531	82.5
For all sustainability items	17.7 (73.8)	9.3 (84.6)	8.3 (63.9)	0.223	51.0

^a^ Positive response defined as selecting ‘agree’ or ‘strongly agree on Likert scale.

*Significant *p* ≤ 0.05, **significant *p* ≤ 0.01.

Interviews confirmed these findings with differences in perceptions of success emerging between each partner group. Academic participants predominantly based their positive views of success on development of the research proposal.

I think it's generally achieved very well, all of these objectives. Certainly with putting in the research proposal, and synthesising the results.

(Academic, 36–45 years, male)

 (…) it [AVONet] enabled us to put together a grant proposal at the end of it, it allowed us to make very good partnerships with people who we wanted to make partnerships.

(Academic, 56–65 years, male)

In contrast, practitioners highlighted general collaborative success and specified practical reasons why the collaborative had been useful for them. These reasons differed to those traditionally perceived as useful in an academic organization, such as aiding the development of services (instead of a research proposal) and providing support for the local community (instead of the publication of research papers).

(…) I found it valuable because it informed me about where I should develop services, and propose to develop services.

(Practitioner, 36–45 years, male)

My personal view is that university life tends to, and can, operate in isolation and I think the strength of this [AVONet] is that, you've got the authority, the knowledge and the evidence, from the university interacting with and supporting the local community

(Practitioner, 56–65 years, male)

Practitioners also stated that AVONet may have been less successful in its level of inclusion of the full range of practitioners. For example, academics were identified as more dominant during collaborative initiation, which may have led to bias towards supporting the needs of academic rather than practitioner contributors.

At the earlier stages it was probably more academic um weighted.

(Practitioner, 36–45 years, male)

… I think maybe having involved some wider partners at an earlier stage [would have contributed to better success] …

(Practitioner, 46–55 years, male)

This was supported by academics who expressed a more positive perception of tangible outcomes, such as a comprehensive literature review and a grant proposal.

(…) having a network makes it look much more serious that we have put a bid in (…)

(Academic, 56–65 years, male)

A comprehensive review and I think we did it and we are now very close to publishing the report of the Avon Network activities.

(Academic, 36–45 years, female)

Despite the less positive responses from practitioners, they did highlight some examples of practical outcomes they had experienced as a result of being a partner of the AVONet.

There's a meeting of about eight or ten people, organisations in [name of city] about whether we can provide a service for, to help people become more physically active in [name of city]. These people I sort of know of or met through the AVONet (…)

(Practitioner, 36–45 years, male)

(…) we've invited both [partner of core management group] and [partner of core management group] up to the national coalition to share this work further afield.

(Practitioner, 56–65 years, male)

Some practitioners emphasized that tangible and practical outcomes over and above networking were necessary for them to remain committed to such a collaborative. Thus, this may serve as a further reason to include practitioners within the CMG at an early stage.

(…) while your line managers ask why you've done that, what you've got out of it, if you can't give them an instant answer then, you know, other than networking, because networking, yes it's valuable but it's not really always what a line manager would want to hear. He'd want to know then what that networking had produced afterwards (…)

(Practitioner, 26–35 years, male)

(…) in terms of how it's helped me on a day to day practical point of view, you know, it still has not done that much.

(Practitioner, 46–55 years, male)

### Structure

Overall, 53% of participant responses to structure questions were positive, although academics provided a higher percentage of positive answers (69 versus 40%) (see Table 2). Overall group differences in responses were significantly different (*U* = 125.5; *p* = 0.001). These between-group differences emerged for overall satisfaction (*U* = 114.5; *p* = 0.011), degree of interdisciplinarity (*U* = 114.0; *p* = 0.013), contribution to meeting individual organizations' aims and objectives (*U* = 107.0; *p* = 0.035) and influence of personal actions (*U* = 107.5; *p* = 0.041). There was a strong positive correlation between the mean structure and success ratings (*r* = 0.756; *p* < 0.001) (see Table 2).

Interviews also revealed that both groups felt a sense of satisfaction with structure, with academics providing more positive and enthusiastic responses. Participants highlighted the importance of the CMG, hierarchical structure and workshops. The structure was perceived to aid relationship building and efficient use of collaborative partners' time.

(…) I think that the core management group are really kind of motivated and driven and are really behind the Avon Network, which is really nice, because like you can feel that there's that kind of, that driving force (…)

(Academic, 36–45 years, female)

(…) I think it's an excellent model because it's got that, it's got all those levels.

(Practitioner, 56–65 years, male)

Constructive criticism of the AVONet from both groups focused on insufficient inclusion of practitioners and policy makers within the CMG from initiation. This could help to explain why academics perceived more positive outcomes from the collaborative.

(…) maybe it should from the very beginning [have] had other agencies involved, and they could have driven it forward together.

(Academic, 36–45 years, female)

(…) what I've not seen quite so many of are what I call the policy makers.

(Practitioner, 56–65 years, male)

### Interactions within AVONet

Sixty-three percent of participants agreed that there was an appropriate balance between information transfer and exchange (see Table 2). The majority (82%) of academics agreed or strongly agreed, compared with 46% of practitioners. Overall group differences in responses were not significant (*U* = 97.5; *p* = 0.134). A strong positive correlation was observed between the mean success rating and perceived balance between information transfer and exchange (*r* = 0.545; *p* = 0.009).

This finding was supported by some of the qualitative results. Academics commented on rewarding and useful interactions with practitioners, such as the selection of partners to work with on further research grant applications, and suggested social reasons for success, such as friendly leadership.

I think having clear leaders with friendly personalities, who are approachable has certainly encouraged the development of relationships.

(Academic, 36–45 years, male)

An outcome has been that we've picked 2 partners that we're going to work more closely with on the grant that we have actually submitted as well (…)

(Academic, 56–65 years, male)

Although there was no statistically significant difference, the quantitative findings indicated that fewer practitioners than academics agreed or strongly agreed that there was an appropriate balance between information transfer and exchange. This was in part supported by the qualitative results. For example, as a critical observation, practitioners referred to the lack of opportunity to build on new connections and interactions between meetings.

(…) I still think that there should be more communication in between the meetings as well to make everybody feel involved (…)

(Practitioner, 26–35 years, male)

I'm not entirely sure what the follow-up has been. For example, we had that meeting in [name of city] where we all suggested what intervention might be wise to go with the research and I don't know what the outcome of that is (…)

(Practitioner, 36–45 years, male)

Participants also discussed that the collaborative had not supported practitioners as much as it might in developing new useful contacts, thus suggesting a need to improve communications with, and networking opportunities for, practitioners.

(…) the relationship building was a good thing. I don't know whether we achieved it successfully with everyone.

(Academic, 36–45 years, female)

(…) Most of the people in the room at the meetings I had already met before from the, from the delivery point of view (…)

(Practitioner, 26–35 years, male)

### Sustainability

Table 2 shows that overall, 74% of participating collaborative partners agreed or strongly agreed that sustaining AVONet would be beneficial for increasing PA in older adults, improving research and the aims of their organizations. Once again academics (85%) were more positive than practitioners (64%). However, Mann–Whitney *U*-tests revealed no significant between-group differences in overall responses (*U* = 51.0; *p* = 0.223).

Qualitative results indicated that there was positive feedback about the sustainability of AVONet from both academics and practitioners, thus supporting the non-significant survey results.

(…) I think it would be a shame to, for it to disperse now when it's … so many people have been able to come together.

(Academic, 36–45 years, female)

Yes, I think it would be good to keep, to have the Avon [network], yes.

(Practitioner, 46–55 years, male)

Suggestions were made regarding format change, such as forming smaller working groups, increasing focus upon service delivery, involving policy makers and budget holders and collaborating with practitioners on successful project bids.

(…) it really could evolve into being more of a knowledge exchange, more of a planning vehicle, collaborative vehicle for delivery.

(Academic, 56–65 years, male)

(…) there is the potential in the future for what I would call sort of smaller working groups.

(Practitioner, 56–65 years, male)

(…) to ensure that key people from a delivery perspective, people who hold purses and monies are also more aware of it and also invited along to it (…)

(Practitioner, 26–35 years, male)

(…) if we were successful with the project bid, we could involve them in that way.

(Academic, 36–45 years, male)

Academics supported a city council-led network and appointment of a dedicated facilitator.

(…) the heart of the network could move to a more community level so the council [could lead] (…) a person would have that role, as the network coordinator (…)

(Academic, 26–35 years, female)

(…) theoretically the two councils [City Councils] should do it.

(Academic, 56–65 years, male)

Barriers to sustainability such as individual organizations' aims and the lack of long-term funding were also discussed.

it [AVONet] existed largely because there's been promises of a prize at the end of it, which has been a research grant (…)

(Academic, 56–65 years, male)

(…) we [universities] are not facilitators of local provision at this point unless it is driven by research because that's our job [doing research].

(Academic, 56–65 years, male)

## DISCUSSION

This study focused on the perceptions of academic and practitioner partners involved in AVONet with regard to the degree of success of the venture in meeting its objectives, the contribution of the structure of the collaborative to the level of success, the degree of helpful communication and the potential for sustainability of the collaborative.

### Success

AVONet objectively did achieve most of its main objectives that were set at the beginning of the collaborative as it produced a comprehensive guide to active ageing for local decision makers, which has been widely disseminated (Stathi *et al.*, 2014), it secured at least temporary funding to sustain the AVONet through the activities of the Avon Primary Care Research Collaborative, and obtained further external funding via a subsequent LLHW grant to conduct a 24-month pilot study called Active, Connected and Engaged neighbourhoods (http://gtr.rcuk.ac.uk/project/97204AAD-A5EA-4145-AE53-5205C60B9F84). Moreover, overall participants perceived the collaborative to have functioned successfully. However, there were differences with practitioners less likely than academics to report within the survey data positive perceptions of success. There was also reporting in interviews that some of the AVONet objectives were not fully consistent with the aims of their organizations. This is likely to have resulted from the collaborative being conceived and set up by the university academics who were co-applicants of the bid for funding. They were perhaps not fully enlightened to the needs of practitioner partners or they may have been more concerned about fulfilling the funder's needs.

The goals paradox states that success of collaboratives can be negatively influenced by both homogeneity and heterogeneity of organizational aims (Vangen and Huxham, 2012). Goals differ as practitioners are often aiming for immediate local public health outcomes and increased access to training and resources, whilst academics are driven by acquisition of research funds, prestigious publications and broader public health policy (Baker *et al.*, 1999; Spoth and Greenberg, 2011). This highlights the potential importance of achieving diverse representation of stakeholders at the formation stage of multi-sector collaborations when the original aims and objectives of the collaborative are being specified, so that all collaborative partners can explicitly acknowledge these joint aims (Vangen and Huxham, 2012).

Reviewing aims at regular intervals could also help to ensure that all stakeholders are able to provide a more equal input into forming the aims and objectives of the collaborative, and that the collaborative is responsive to the changing needs of collaborative partners throughout the collaboration process. Indeed, the need to acknowledge the goals of each organization within a collaboration forms part of the principles of practice within the academic–practice collaborations proposed by Baker *et al.* (Baker *et al.*, 1999).

### Structure

The structure of AVONet was broadly perceived as successful and related to the overall perceptions of success of the collaborative. However, academics showed more positive responses than practitioners within both the survey and interview data. Practitioners reported the inclusion of AVONet's CMG as a limitation. It is likely that this was due to the dominance of academic stakeholders in the CMG, thus a focus on research may have restricted the amount of communication that was directly helpful to practitioners. A previous qualitative evaluation of 34 NHS cancer networks in England showed success to be associated with a CMG dedicated to maintaining communication in between meetings (Richardson *et al.*, 2005). Further to this, Provan and Kenis (Provan and Kenis, 2008) proposed a theory which outlined three forms of organizational governance: (i) shared governance whereby all partners have equal input; (ii) governance by a lead organization and (iii) governance by a CMG. They proposed that in order for governance by a CMG to be successful, there must be a high goal consensus among collaborative partners. This highlights a possible limitation to successful collaboration within the AVONet, as local practitioners and third sector workers are likely to have different aims to academics (Spoth and Greenberg, 2011). The relaxation or removal of boundaries, such as structural characteristics that may limit communication between academics and practitioners, through the inclusion of practitioners in the CMG from the collaborative design (and grant application) stage could increase the practical focus of future collaborative outcomes (Glendinning, 2003).

### Information transfer and exchange

Positive perceptions of the balance between information transfer and exchange were observed within the AVONet. This is supported by the principles of practice for research collaboratives between academics, practitioners and the community, outlined by Baker *et al.* (Baker *et al.*, 1999). These principles of practice suggest that when creating a community-based research project involving such collaborations, academics should relinquish some control to allow input from practitioners and the community to shape future initiatives including participation in research activities, such as helping to write grant proposals. Partners should also invest time in regular information sharing and communication to facilitate the development of relationships based on mutual respect and trust (Baker *et al.*, 1999). This could be facilitated through a ‘bottom-up’ approach, whereby all parties provide equal input (Glendinning, 2003; Rummery and Coleman, 2003). Thus, this may require a more flexible approach from funders to allow changes and developments in collaborative objectives and activities after the funding has been awarded.

### Sustainability

AVONet partners emphasized that sustaining the collaborative could be beneficial within both the survey and interview data. The suggested strategies for sustainability of AVONet all involve some form of capacity building, such as changes to the collaborative objectives and structure acquisition of resources to help improve the scope for longevity of the collaborative. Allowing the AVONet to respond to these suggestions could lead to the collaborative developing into a network, with communication and information exchange as its focus. Examples included the formation of smaller working groups and practitioner governance. Inclusion of professionals in the governance process has been associated with increased legitimacy in the community in a longitudinal evaluation of a US-based mental health network (Provan *et al.*, 2004). Studies exploring collaborative governance identify smaller working groups as a feasible collaborative structure, which often emerge as and when new tasks need to be completed, suggesting that these groups could be informally integrated into the collaborative structure (Huxham *et al.*, 2000). The inclusion of practitioners in the AVONet's CMG could help to maintain the motivation of practitioners by ensuring that joint aims are established, whilst smaller working groups which emerge in response to the tasks that need to be completed within the collaborative could facilitate more efficient collaborative functioning (Huxham *et al.*, 2000). However, the relationship with and accountability to the collaborative should be made clear (Huxham *et al.*, 2000).

### Strengths and limitations

The use of a mixed-methods design provides diverse sources of information and a more comprehensive understanding of the emerging themes and offers the opportunity to confirm or reject findings (Creswell and Plano Clark, 2011). However, a limitation is that the retrospective data collection might introduce potential bias as a result of selective recall and therefore limit the inference of causality in associations. Longitudinal design, which documents partner reactions and feedback at several time points from inception to sustainability phases, should be considered in future research.

This study was not funded by an external funding body and was conducted independently and upon completion of the AVONet grant. The lead researcher (H.J.L.) worked as an administrator throughout the 10 months of collaborative funding, and there is a possibility that this may have introduced a positive bias to interpretation of the qualitative data. Conversely, the extensive experience with the collaborative and the partners may have provided extra insight.

Future studies evaluating the success of collaboratives could benefit from incorporating a wider variety of methods, such as social network analysis to investigate the collaborative structure in more detail and to explore how structure relates to agency and interactions between collaborative partners (Giddens, 1984; Hawe and Ghali, 2008). Future studies would also benefit from investigating reasons for non-response and from seeking feedback by older adult lay representatives regarding the success of such collaboratives.

## CONCLUSIONS

The AVONet multi-sectoral collaborative can be perceived as successful in terms of meeting its objectives, the contribution of collaborative structure to success, the degree of helpful communication and the potential for sustainability of the collaborative. However, concerns over misaligned aims of academic and practitioner partners and a lack of long-term funding were perceived as possible threats to the sustainability of the collaborative. The relevance of these findings is demonstrated by strong calls from local commissioners to ground their decision-making in evidence and demands of UK research councils for public health research to be multidisciplinary, multi-agency, feature user involvement and show direct impact on policy and practice (Kelly and McNicoll, 2011). Thus, exploring the determinants of success of collaboratives as seen by their contributors is particularly pertinent. Suggestions arising from these results that may enhance the sustainability of multi-sectoral collaboratives and facilitate the achievement of their aims and objectives include the following.

- *Core management group*: Collaboratives should be governed by a fully representative CMG from initiation.
- *Inclusion*: Accessible meetings, communication aligned with language of each organization and a dedicated facilitator should be utilized to promote relationship building.
- *Aims*: Practitioners and end users should be involved from initiation to develop common goals, which are reviewed at regular, pre-determined time intervals.
- *Smaller working groups*: Small, informal working groups should be established.
- *Funding*: Funding for collaboratives of academics and health practitioners with a broad set of aims that include service delivery and evaluation should be encouraged.

## FUNDING

The Avon Ageing Network was supported by the Lifelong Health and Well-being Initiative-Phase 2 (grant number G0900040). Lifelong Health and Well-being is led by the Medical Research Council on behalf of the Arts and Humanities Research Council; Biotechnology and Biological Sciences Research Council; Economic and Social Research Council and Engineering and Physical Sciences Research Council in partnership with the four UK health departments. Funding to pay the Open Access publication charges for this article was provided by the University of Bath.
